# Celebrating milestones in cell communication and signaling: Forthcoming joint ICCNS–ARBIOCOM anniversary meeting

**DOI:** 10.1002/ccs3.70052

**Published:** 2025-10-17

**Authors:** Brahim Chaqour, Annick Perbal, Bernard Perbal

**Affiliations:** ^1^ Dept of Molecular Ophthalmology, Perelman School of Medicine University of Pennsylvania Philadelphia Pennsylvania USA; ^2^ International CCN Society Nice France

**Keywords:** CCN proteins, cell communication, conference meeting, signaling, signalomics

## Abstract

The Joint Meeting commemorating the 25th anniversary of the International CCN Society (ICCNS) and the 5th anniversary of the Association for Research on Biosignaling and Communication (ARBIOCOM) will take place on December 13–17, 2025, in Nice, France. This multidisciplinary forum will bring together researchers in molecular and cellular biology, systems biology, translational medicine, and related fields to explore advances in intercellular communication and signal transduction. The program will include keynote lectures, thematic sessions, and attendee presentations spanning juxtacrine and paracrine interactions, extracellular matrix‐mediated signaling with emphasis on CCN proteins, endocrine and neuroendocrine regulation, immune‐modulatory circuits, extracellular vesicle biology, and systemic regulation by soluble factors. A special focus will address how dysregulated signaling drives cancer, musculoskeletal, cardiovascular, neurodegenerative, and developmental disorders. Sessions will encourage contributions using single‐cell and spatial transcriptomics, dynamic proteomics, and systems‐level approaches to map signaling dynamics in vivo. The meeting will also highlight pivotal role of the *Journal of Cell Communication and Signaling* (*JCCS*), the official ICCNS journal published by Wiley, as a leading platform for disseminating impactful research in biosignaling. This joint anniversary event will provide an international venue for advancing knowledge, fostering collaborations, and shaping the future of research in cell communication and signaling.

## Graphical abstract

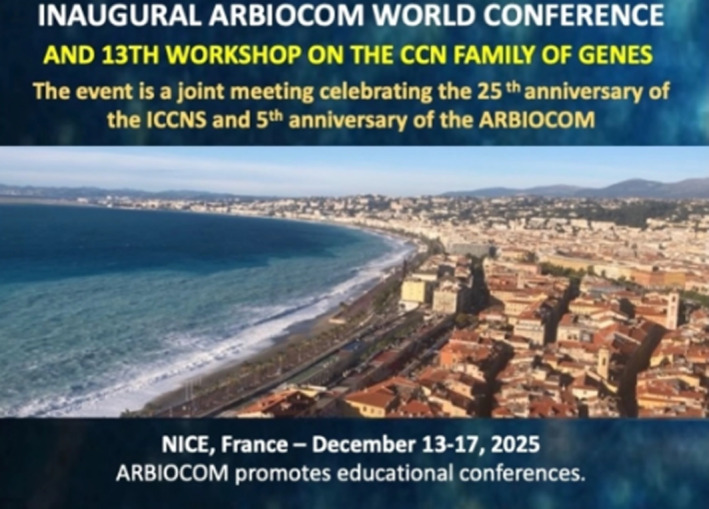

## 1 EDITORIAL

It is with great enthusiasm that we invite the broad scientific community to join us for a truly special event: the Joint Meeting Celebrating the 25th Anniversary of the International CCN Society (ICCNS) and the 5th Anniversary of the ARBIOCOM Network, to be held in beautiful Nice, France, on the French Riviera. This landmark gathering will bring together leading researchers, clinicians, and innovators from around the world to share cutting‐edge discoveries, foster new collaborations, and reflect on decades of progress in the study of cell communication networks, extracellular and intracellular signaling, and their impact on health and disease.

Over the past 25 years, the ICCNS in partnership with JCCS has been at the forefront of advancing research on all direct and remote mediators of cell communication and their diverse roles in development, tissue repair, cancer, and regenerative medicine among other areas. In parallel, ARBIOCOM has, over its 5 years of activity, provided a unique platform for researchers, clinician‐scientists, and representatives from academic institutions, biomedical industries, and scientific societies to share their research and views with a broad audience focused on advancing our understanding of biosignaling and cellular communication networks, with applications in drug discovery and disease treatment. Together, these two communities have built strong foundations that continue to expand the boundaries of cell signaling research.

This premier conference will feature experts as well as emerging voices from across the life sciences. The program will explore both local and remote modes of cellular communication, the extracellular matrix including the matricellular CCN proteins, soluble circulating growth and inflammatory factors, hormones, and extracellular vesicles, in both health and disease. This meeting offers the opportunity to presenters to discuss a wide range of topics from metabolic and circadian regulation of signaling pathways, to endocrine, immune, and neuronal signaling (e.g., senescence, efferocytosis, and oncogenesis among other areas). The conference is also open to exploring temporal and spatial proteomics dynamics as well as comprehensive spatial transcriptomics, offering a forward‐looking perspective on large‐scale, translational, whole‐organism approaches.

We are also proud to emphasize the pivotal role of the *Journal of Cell Communication and Signaling* (*JCCS*), the official journal of the ICCNS, in disseminating high‐impact research. JCCS has been instrumental in shaping the field, and this meeting will highlight its contributions while providing participants with opportunities to discuss new avenues for scholarly publication and knowledge exchange. This conference provides an excellent opportunity to honor individuals who have played a vital role in the editorial and review process by presenting them with symbolic honorary plaques in recognition of their dedication and contributions. In addition, a congratulatory plaque will be given to the authors of the most‐cited JCCS publication as a tribute to their impactful work. Each year, new individuals will be selected and recognized, ensuring that outstanding contributions continue to be acknowledged.

Your participation and involvement will help us mold this initiative into a world‐class platform that unites the efforts of researchers from diverse scientific backgrounds, societies, and organizations to accelerate progress in all areas of cell communication and signaling. Pre‐registration is now open at https://arbiocom.wordpress.com, with registration packages including lodging, meals, and social events.

Whether your goals are fundamental or clinical research, innovation, discovery, clinical partnerships, licensing, publishing, industry collaborations, or commercialization, this joint anniversary meeting will provide an outstanding opportunity to advance your work and connect with a vibrant international community. We warmly encourage scientists at all career stages to register and participate in this exceptional event as we celebrate past achievements, highlight present advances, and shape the future of cell communication and signaling research.

## AUTHOR CONTRIBUTIONS

All authors have equally contributed to the preparation of the editorial.

## CONFLICT OF INTEREST STATEMENT

The authors declare no conflicts of interest.

## Data Availability

Data sharing not applicable to this article as no datasets were generated or analyzed during the current study.

